# Structure of a highly acidic β-lactamase from the moderate halophile *Chromohalobacter* sp. 560 and the discovery of a Cs^+^-selective binding site

**DOI:** 10.1107/S1399004714027734

**Published:** 2015-02-26

**Authors:** Shigeki Arai, Yasushi Yonezawa, Nobuo Okazaki, Fumiko Matsumoto, Chie Shibazaki, Rumi Shimizu, Mitsugu Yamada, Motoyasu Adachi, Taro Tamada, Masahide Kawamoto, Hiroko Tokunaga, Matsujiro Ishibashi, Michael Blaber, Masao Tokunaga, Ryota Kuroki

**Affiliations:** a Quantum Beam Science Directorate, Japan Atomic Energy Agency, 2-4 Shirakata-shirane, Tokai, Ibaraki 319-1195, Japan; b Saga Prefectural Regional Industry Support Center, Kyushu Synchrotron Light Research Center, 8-7 Yayoigaoka, Tosu, Saga 841-0005, Japan; c Applied and Molecular Microbiology, Faculty of Agriculture, Kagoshima University, 1-21-24 Korimoto, Kagoshima 890-0065, Japan; d College of Medicine, Florida State University, 1115 West Call Street, Tallahassee, FL 32306-4300, USA

**Keywords:** β-lactamase, *Chromohalobacter* sp., Sr^2+^ binding, Cs^+^ binding

## Abstract

Environmentally friendly absorbents are needed for Sr^2+^ and Cs^+^, as the removal of the radioactive Sr^2+^ and Cs^+^ that has leaked from the Fukushima Nuclear Power Plant is one of the most important problems in Japan. Halophilic proteins are known to have many acidic residues on their surface that can provide specific binding sites for metal ions such as Cs^+^ or Sr^2+^. The crystal structure of a halophilic β-lactamase from *Chromohalobacter* sp. 560 (HaBLA) was determined to resolutions of between 1.8 and 2.9 Å in space group *P*3_1_ using X-ray crystallography. Moreover, the locations of bound Sr^2+^ and Cs^+^ ions were identified by anomalous X-ray diffraction. The location of one Cs^+^-specific binding site was identified in HaBLA even in the presence of a ninefold molar excess of Na^+^ (90 m*M* Na^+^/10 m*M* Cs^+^). From an activity assay using isothermal titration calorimetry, the bound Sr^2+^ and Cs^+^ ions do not significantly affect the enzymatic function of HaBLA. The observation of a selective and high-affinity Cs^+^-binding site provides important information that is useful for the design of artificial Cs^+^-binding sites that may be useful in the bioremediation of radioactive isotopes.

## Abstract 2

The tertiary structure of a β-lactamase derived from the halobacterium *Chromohalobacter* sp. 560 (HaBLA) was determined by X-ray crystallography. Three unique Sr^2+^-binding sites and one Cs^+^-binding site were discovered in the HaBLA molecule.

## 1. Introduction   

Proteins derived from extreme halophiles and extracellular and periplasmic fractions of moderate halophiles have highly acidic protein surfaces (owing to an abundant content of acidic amino acids; Rao & Argos, 1981 ▶; Pieper *et al.*, 1998 ▶) that may interact with metal ions. As of November 2014, 12 types of halophilic proteins have been deposited in the Protein Data Bank (PDB) with a ratio of acidic to basic amino acids [(Glu + Asp)/(Lys + Arg + His)] of greater than 1.5. The coordinates of these halophilic proteins include various types of specifically bound metal ions such as Na^+^, Mg^2+^, K^+^, Ca^2+^, Fe^3+^, Zn^2+^, Sr^2+^ and Cd^2+^, which contribute to the activity and structural stability of the enzymes. For example, alkaline phosphatases from *Halobacterium salinarum* (HsAP; PDB entry 2x98) and from *Halomonas* sp. 593 (HaAP; PDB entry 3wbh) require Na^+^ for quaternary-structure formation and Mg^2+^ and Zn^2+^ for enzyme activity (Ishibashi *et al.*, 2005 ▶, 2011 ▶; Wende *et al.*, 2010 ▶; Arai *et al.*, 2014 ▶). Nucleoside diphosphate kinase derived from *H. salinarum* (HsNDK; PDB entry 2az1) requires Mg^2+^ or Ca^2+^ for the recognition of a nucleoside as a substrate (Besir *et al.*, 2005 ▶). In addition, a 50S ribosomal subunit derived from *Haloarcula marismortui* (PDB entry 1yhq) requires Na^+^, Mg^2+^, K^+^, Sr^2+^ and Cd^2+^ for stabilization of the subunit conformation (Tu *et al.*, 2005 ▶; Blaha *et al.*, 2008 ▶).

Because various metal ion-binding sites exist in halophilic proteins, novel metal ion-binding sites with an affinity for harmful metals and rare metals could potentially be identified using X-ray crystallographic analysis in the presence of such metal ions. If so, the structures can be used as scaffolds for constructing artificial binding sites for metal ions that could serve as environmentally friendly nontoxic protein-based specific metal-ion absorbents.

Absorbents are particularly needed for Sr^2+^ and Cs^+^, as the removal of the radioactive Sr^2+^ and Cs^+^ that leaked from the Fukushima Dai-ichi Nuclear Power Plant is one of the most important social problems in Japan. Most radioactive Cs^+^ deposited on the land at Fukushima (2.4 PBq) is fixed in soil particles, mainly weathered biotite (Mukai *et al.*, 2014 ▶). Removal of the contaminated soil has now been carried out (Sato, 2011 ▶). However, this decontamination method is high-cost, and disposal of the removed soil is a problem (IAEA, 1994 ▶; Vidal *et al.*, 2001 ▶). In addition, there is no effective method to collect the radioactive Cs^+^ released gradually from the remaining soil in the environment over the long term.

Protein absorbents may have several merits for the collection of harmful or rare metal ions. For example, protein absorbents may be available for the improvement of phyto­remediation. If protein absorbents for Cs^+^ are expressed in specific parts of plants such as roots, flowers or seeds by gene recombination, Cs^+^ might be accumulated in these parts. This application will contribute to reducing radioactive waste. Moreover, plants with protein absorbents will capture the Cs^+^ released gradually from soil particles over the long term.

To evaluate metal-ion affinity and to identify protein metal-binding sites for Sr^2+^ and Cs^+^, a class C β-lactamase (BLA) derived from the moderate halophile *Chromohalobacter* sp. 560 (HaBLA; molecular weight 39 536) was chosen as a model system. This selection was based on the following reasons.

(i) Abundant content of acidic amino acids. HaBLA, although a moderately halophilic periplasmic protein, has a particularly abundant content of acidic amino acids similar to those in extremely halophilic proteins. The ratio of acidic to basic amino acids in HaBLA [(Glu + Asp)/(Lys + Arg + His) = 57/32] is significantly higher than those in homologous non­halophilic class C BLAs derived from *Burkholderia* sp. Ch1-1 [sequence identity 50.0%, (Glu + Asp)/(Lys + Arg + His) = 31/40] and *Serratia marcescens* [sequence identity 52.0%, (Glu + Asp)/(Lys + Arg + His) = 28/40]. With this abundant content of acidic amino acids, the HaBLA molecule may provide scaffolds for metal ion-binding sites.
(ii) High solubility and mesophilic heterologous expression. HaBLA shows high solubility, which results from the high content of acidic amino acids, which prevents the aggregation of the HaBLA molecules (Tokunaga *et al.*, 2004 ▶; Tokunaga, Arakawa *et al.*, 2010 ▶; Tokunaga, Saito *et al.*, 2010 ▶). Furthermore, despite the halophilic character of HaBLA, it can be efficiently expressed in *Escherichia coli* cytoplasm in a fully active form (Tokunaga *et al.*, 2006 ▶).

To our knowledge, this is the first report of the tertiary structure of a halophilic BLA. Here, we report the discovery of specific binding sites for Sr^2+^ and Cs^+^ in the HaBLA molecule using X-ray crystallography.

## 2. Materials and methods   

### 2.1. Expression and purification of HaBLA   

Wild-type HaBLA (WT-HaBLA) has Asn-Gly dipeptide sequences at residue positions 288–289 and 321–322 which can promote deamidation. Because deamidation often causes heterogeneity of protein molecules in crystals and deteriorates the diffraction resolution, the N288Q/N321Q mutant (NQ-HaBLA) was prepared and used as the standard HaBLA in this study.

Recombinant expression of the HaBLA proteins was performed essentially as described previously (Tokunaga *et al.*, 2004 ▶, 2006 ▶). In brief, HaBLA was expressed as an amino-terminally His-tagged fusion protein in *E. coli* BL21 Star (DE3) cells. The HaBLA gene was inserted into the pET-15b His-tag fusion vector (Novagen, Wisconsin, USA) to construct pET-15b-bla. The expressed HaBLA proteins were purified to homogeneity using an Ni–IMAC column (PhyNexus Inc., California, USA); subsequently, the His tag of HaBLA was removed by thrombin digestion. The resulting non-His-tagged HaBLA protein was purified using a Resource Q anion-exchange column (GE Healthcare, New Jersey, USA).

### 2.2. Crystallization of HaBLA   

Initial screening for crystallization conditions for WT-HaBLA and NQ-HaBLA was performed by the sitting-drop vapour-diffusion method using a 96-well Intelli-Plate (Hampton Research, California, USA) and a Hydra II Plus One crystallization workstation (Matrix Technologies, New Hampshire, USA) at 293 K. Before crystallization, HaBLA was dialyzed against 50 m*M* HEPES buffer pH 7.0 containing 200 m*M* NaCl. Sitting drops were prepared by mixing 0.3 µl each of protein solution and reservoir solution and the resulting drop was equilibrated against 70 µl reservoir solution. The initial search for crystallization conditions was performed using commercially available precipitant solutions including PEG/Ion, PEG/Ion 2, Crystal Screen, Crystal Screen 2 (Hampton Research, California, USA) and Wizard Screen I and II (Emerald Bio, Washington, USA). After initial screening identified useful crystallization conditions, the protein concentration and precipitant conditions were optimized to obtain diffraction-quality crystals.

The WT-HaBLA crystal was grown in 50 m*M* HEPES buffer pH 7.0 containing 200 m*M* NaCl, 200 m*M* magnesium formate, 30%(*w*/*v*) polyethylene glycol (PEG) 3350 (which originated from PEG/Ion solution No. 20) with 47 mg ml^−1^ protein at 293 K. The NQ-HaBLA crystals were grown in 100 m*M* MES buffer pH 6.5 containing 200 m*M* calcium acetate, 18%(*w*/*v*) PEG 8000 (which originated from Crystal Screen solution No. 46) with 30 mg ml^−1^ protein at 293 K.

The obtained NQ-HaBLA crystals were soaked in five different solutions containing Cs^+^ or Sr^2+^ in order to test their affinities for these ions: solutions 1A, 1B and 1C contained NaCl/CsCl at 0/100, 75/25 and 90/10 m*M*, respectively (in order to compare the affinity between Na^+^ and Cs^+^), while solutions 2A and 2B contained calcium acetate/strontium acetate at 0/200 and 100/100 m*M*, respectively, in order to compare the affinity between Ca^2+^ and Sr^2+^.

### 2.3. Diffraction data collection and structure determination   

X-ray diffraction data collection was carried out on beamlines BL5A, BL17A, NE3A and NW12A at the Photon Factory (PF), Tsukuba, Japan, beamlines BL38B1 and BL41XU at SPring-8, Hyogo, Japan and beamline BL7 at the SAGA Light Source (SAGA-LS). All crystals [WT-HaBLA crystals and NQ-HaBLA crystals without soaking and with soaking in conditions 1A (0 m*M* Na^+^/100 m*M* Cs^+^), 1B (75 m*M* Na^+^/25 m*M* Cs^+^), 1C (90 m*M* Na^+^/10 m*M* Cs^+^), 2A (0 m*M* Ca^2+^/200 m*M* Sr^2+^) and 2B (100 m*M* Ca^2+^/100 m*M* Sr^2+^)] were measured using a wavelength of 1.000 Å. To identify the presence of Cs^+^ by anomalous diffraction, wavelengths of 2.066 and 2.166 Å were used for crystals from conditions 1A, 1B and 1C (Table 1 ▶). In addition, to identify Sr^2+^ by anomalous diffraction, a wavelength of 0.769 Å was used for the crystals from conditions 2A and 2B (see §[Sec sec2.5]2.5).

All data sets were collected at 100 K and the crystals were cryoprotected with NVH oil (Hampton Research, California, USA). Diffraction data were integrated and scaled using the *HKL*-2000 suite of programs (Otwinowski & Minor, 1997 ▶). The WT-HaBLA crystal diffracted to 2.9 Å resolution (Table 1 ▶). The initial phase information for WT-HaBLA was obtained by the molecular-replacement (MR) method using *MOLREP* (Vagin & Teplyakov, 2010 ▶) and the nonhalophilic class C BLA (EaBLA) from *Enterobacter aerogenes* (PDB entry 1zkj, sequence identity 44%) as the search model (Kim *et al.*, 2006 ▶).

The initial phase information for nonsoaked NQ-HaBLA was obtained using the rigid-body refinement method with WT-HaBLA as the search model. The initial phase information for NQ-HaBLA in conditions 1A, 1B, 1C, 2A and 2B was obtained by rigid-body refinement using the structure determined from nonsoaked NQ-HaBLA as the search model. Model building and refinement were performed using *Coot* (Emsley & Cowtan, 2004 ▶), *CNS* v.1.21 (Brünger *et al.*, 1998 ▶) and *REFMAC*5 (Murshudov *et al.*, 2011 ▶). All data sets exhibited good overall completeness and the refined structures exhibited acceptable values for the stereochemistry and crystallographic residuals (Table 1 ▶). According to *RAMPAGE* (Lovell *et al.*, 2003 ▶), the final model obtained for WT-HaBLA had 88.8% of the residues in favoured conformations in the Ramachandran plot. For NQ-HaBLA without soaking and NQ-HaBLA in conditions 1A, 1B, 1C, 2A and 2B, 90.5, 91.2, 90.5, 91.3, 91.1 and 89.3% of the residues were in favoured conformations, respectively. The r.m.s. deviations of the atoms in the BLAs for structural comparison were calculated using *LSQKAB* in the *CCP*4 package (Winn *et al.*, 2011 ▶) and the web-based program *PDBeFold* (http://www.ebi.ac.uk/msd-srv/ssm/; Krissinel & Henrick, 2004 ▶). The molecular volume of each BLA was calculated using the web-based program 3*V* (Voss & Gerstein, 2010 ▶).

### 2.4. Identification of metal ion-binding sites   

The metal ions in the crystal structures of WT-HaBLA and NQ-HaBLA were identified by evaluation of electron density, coordination geometry and ionic bond distances. The distributions of the ionic bond distance between the acceptor and the putative metal ion (2.34–2.81 Å for Na—O, 1.92–2.31 Å for Mg—O, 2.35–2.76 Å for Ca—O, 2.53–2.86 Å for Sr—O and 3.02–3.30 Å for Cs—O) calculated from ionic radii (Haynes *et al.*, 2013 ▶; Jia, 1991 ▶; Shannon, 1976 ▶) were included as criteria to identify metal-atom species. Tetrahedral or octahedral co­ordination for Na^+^ and Mg^2+^ and octahedral or cubic coordination for Ca^2+^, Sr^2+^ and Cs^+^ were searched for as the ideal geometries of acceptor atoms according to Pauling’s rules (Pauling, 1929 ▶).

The procedure used to identify the metal ions in the crystal structures was as follows. To identify Ca^2+^, Sr^2+^ and Cs^+^, locations showing *F*_o_ − *F*_c_ difference electron density of greater than 2.5σ after placing a water (O atom) and less than 0.5σ after placing Ca^2+^, Cs^+^ or Sr^2+^ ions were first identified. The appropriate metal atoms for the *F*_o_ − *F*_c_ map were then added to the HaBLA structure. Next, it was determined whether the arrangement of acceptor atoms including solvent around the placed metal ions was appropriate for metal coordination. Since the electron densities of Na^+^ and Mg^2+^ are similar to that of the O atom of water, identification of Na^+^ and Mg^2+^ was attempted by the evaluation not of electron density but of the ionic bond distances and coordination geometries.

### 2.5. Anomalous diffraction data analysis for distinguishing metal ions   

To identify Cs^+^ in condition 1A (0 m*M* Na^+^/100 m*M* Cs^+^), in condition 1B (75 m*M* Na^+^/25 m*M* Cs^+^) and in condition 1C (90 m*M* Na^+^/10 m*M* Cs^+^) and to identify Sr^2+^ in condition 2A (0 m*M* Ca^2+^/200 m*M* Sr^2+^) and in condition 2B (100 m*M* Ca^2+^/100 m*M* Sr^2+^), anomalous diffraction data were collected. Using X-ray absorption spectroscopy analysis (Supplementary Fig. S1), the wavelength for anomalous diffraction data collection to identify Cs^+^ was determined to be λ = 2.066 Å for BL17A at PF and λ = 2.166 Å for BL7 at SAGA-LS, which are slightly shorter than the absorption-edge wavelength of Cs (λ = 2.175 Å). In addition, the wavelength for anomalous diffraction data collection to identify Sr^2+^ was determined to be λ = 0.769 Å for NW12A and BL5A at PF and BL38B1 at SPring-8, which is slightly shorter than the absorption-edge wavelength of Sr (λ = 0.770 Å). The calculated phase (ϕ_c_) parameter (Read, 1986 ▶) derived from the crystal structure of NQ-HaBLA without soaking was added to the anomalous diffraction data from conditions 1A, 1B, 1C, 2A and 2B using *SFALL* (Agarwal, 1978 ▶) from the *CCP*4 package, and the anomalous difference Fourier maps of NQ-HaBLA in conditions 1A, 1B, 1C, 2A and 2B were calculated using *SIGMAA* (Read, 1986 ▶) and *FFT* (Immirzi, 1966 ▶) from the *CCP*4 package.

### 2.6. Enzymatic assay   

An enzymatic assay of HaBLA was performed using a VP-ITC (MicroCal Inc., Massachusetts, USA) isothermal titration calorimeter (ITC). In all ITC studies the syringe solution was 5 m*M* benzylpenicillin (Penicillin G; Wako Pure Chemical Industries, Japan), a substrate utilized by HaBLA. The sample cell (1.4 ml) contained 0.35 µ*M* HaBLA solution. The reaction medium consisted of 0.1 *M* HEPES–Tris buffer pH 7.4 with or without metal chlorides (0.1 *M* NaCl, 0.5 *M* NaCl, 1.0 *M* NaCl, 2.0 *M* NaCl, 4.0 *M* NaCl, 1.0 *M* CsCl, 1.0 *M* NaCl/40 m*M* MgCl_2_, 1.0 *M* NaCl/40 m*M* CaCl_2_ or 1.0 *M* NaCl/40 m*M* SrCl_2_). After a 60 s baseline had been recorded, the titration reactions were performed by sequential injections of 20 µl penicillin G solution into the sample cell. The duration of the injection was 10 s. The syringe was rotated at 307 rev min^−1^ at 298.15 K. Data used for analysis were taken from 30 s after the start of injection until the power (*P*) generated by the reaction returned to the baseline. Triplet measurements were collected in each case.

A brief description of the equations utilized for ITC analyses (Todd & Gomez, 2001 ▶) follows. The thermal power *P* generated by the reaction is directly measured for the enzymatic reaction, which is given as the heat (*Q*) measured as a function of time (d*t*), 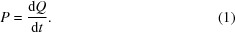
The relationship between the enzymatic reaction rate (*R*_*t*_) and *P* is expressed by
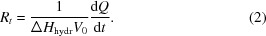

*V*_0_ is the volume of the sample cell (1.4 ml). Δ*H*_hydr_ is the total molar enthalpy for the reaction, which is found by integrating the change of heat with time, 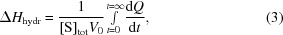
where [*S*]_tot_ is the molar concentration of the substrate being hydrolysed. The substrate concentration at any time point ([*S*]_*t*_) during the enzymatic reaction is calculated from
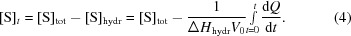
[S]_hydr_ is the substrate concentration that has been hydrolysed at any time point during the enzymatic reaction. From the measured d*Q*/d*t* and (2), (3) and (4), a continuous set of ([S]_*t*_, *R*_*t*_) data points is obtained. A continuous set of ([S]_*t*_, *R*_*t*_) is fitted by the Michaelis–Menten equation (5) in *Origin* 7.0 (MicroCal Inc., Massachusetts, USA) in order to obtain *K*_cat_ and *K*_m_ values, 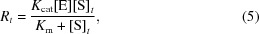
where [E] is the enzyme concentration in the sample cell.

## 3. Results   

### 3.1. Overall structure of wild-type and N288Q/N321Q mutant HaBLAs   

**Figure 1:**
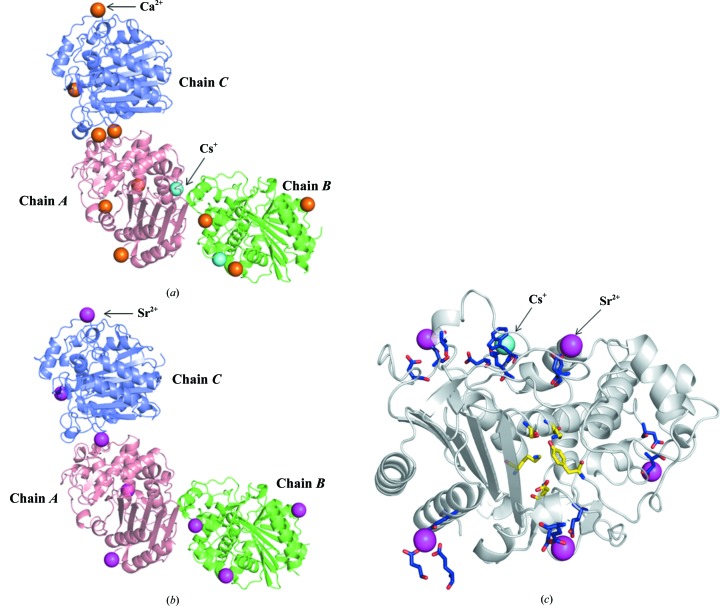
The overall structure of NQ-HaBLA. The asymmetric units of NQ-HaBLA from (*a*) condition 1B (75 m*M* Na^+^/25 m*M* Cs^+^) and (*b*) condition 2A (0 m*M* Ca^2+^/200 m*M* Sr^2+^) are shown. Chains *A*, *B* and *C* are coloured red, blue and green, respectively. The orange, magenta and cyan spheres show Ca^2+^, Sr^2+^ and Cs^+^, respectively. (*c*) Sr^2+^-bound and Cs^+^-bound structure of NQ-HaBLA, in which Sr^2+^ and Cs^+^ observed around chains *A*, *B* and *C* in condition 1B and condition 2A are integrated into a single molecule of NQ-HaBLA. The residues recognizing Sr^2+^ and Cs^+^ are shown as blue sticks. The active-site residues are shown as yellow sticks.

The crystallization of WT-HaBLA and NQ-HaBLA required divalent metal ions (Mg^2+^ or Ca^2+^). Both WT-HaBLA and NQ-HaBLA crystallized isomorphously, and both contained three HaBLA molecules in the asymmetric unit. The overall structure of Ca^2+^-bound and Cs^+^-bound NQ-HaBLA from condition 1B (75 m*M* Na^+^/25 m*M* Cs^+^) determined at 1.8 Å resolution and Sr^2+^-bound NQ-HaBLA from condition 2A (0 m*M* Ca^2+^/200 m*M* Sr^2+^) determined at 1.9 Å resolution are shown in Fig. 1 ▶.

**Figure 2:**
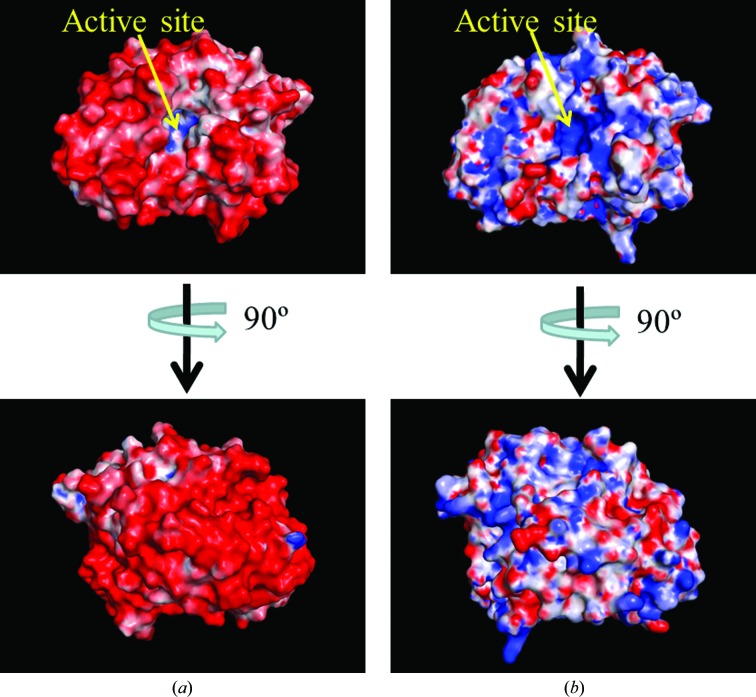
Comparison of the electrostatic potentials of the molecular surfaces of (*a*) WT-HaBLA and (*b*) nonhalophilic BLA from *E. aerogenes* used for molecular replacement (EaBLA; PDB entry 1zkj). The electrostatic potential is shown in the range −20 *kT* e^−1^ (red) to 20 *kT* e^−1^ (blue). This figure was created using the *APBS* plugin (Baker *et al.*, 2001 ▶) in *PyMOL* (http://www.pymol.org).

WT-HaBLA crystallized in the presence of Na^+^ and Mg^2+^ but without Sr^2+^ and Cs^+^. The tertiary structure of WT-HaBLA was determined to 2.9 Å resolution (Table 1 ▶). The solvent content in the WT-HaBLA crystal was 44.6%. The tertiary structure of WT-HaBLA contains 12 α-helices and 16 β-strands, which is the same as in halophilic class C BLAs. The root-mean-square deviations (r.m.s.d.s) for C^α^ atoms between the monomers of WT-HaBLA and the non­halophilic EaBLA (PDB entry 1zkj) chosen as the search model for molecular replacement are less than 1.2 Å. The surface charge distribution of the WT-HaBLA molecule is shown in Fig. 2 ▶(*a*) and is compared with that of the EaBLA. The molecular surface of WT-HaBLA is almost fully occupied by negative charges, whereas equivalent positive and negative charges appear on the molecular surface of EaBLA, as shown in Fig. 2 ▶(*b*).

NQ-HaBLA was initially crystallized with Na^+^ and Ca^2+^ and the structure was determined to 1.8 Å resolution. The solvent content in the NQ-HaBLA crystal is 43.0%. The r.m.s.d. on C^α^ atoms between the monomers of WT-HaBLA and NQ-HaBLA are less than 0.5 Å; therefore, the N288Q/N321Q mutation did not significantly alter the overall structure of HaBLA.

### 3.2. Active-site structure of HaBLA   

**Figure 3:**
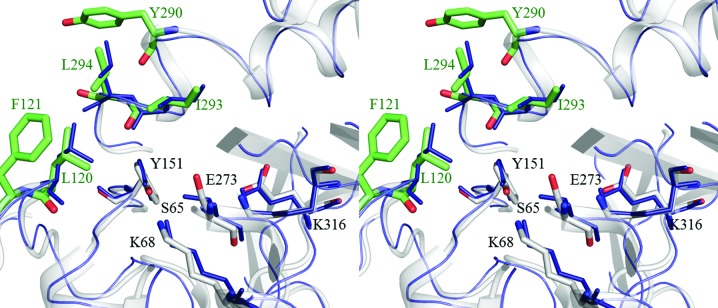
Relaxed stereoview of the catalytic residues (grey sticks) and hydrophobic cluster (green sticks) of WT-HaBLA. The structure of EaBLA (blue) is superposed based on the catalytic residues (Ser65, Lys68, Tyr151, Glu273 and Lys316 of HaBLA). The side chains are omitted apart from those of the residues of the catalytic site and the hydrophobic cluster.

Ser64, Lys67, Tyr150, Glu272 and Lys315 are involved in the catalytic action of nonhalophilic class C BLAs (Powers & Shoichet, 2002 ▶; Beadle & Shoichet, 2002 ▶; Hata *et al.*, 2005 ▶; Drawz *et al.*, 2010 ▶). These residues are conserved as Ser65, Lys68, Tyr151, Glu272 and Lys312 in EaBLA and as Ser65, Lys68, Tyr151, Glu273 and Lys316 in HaBLA. From a structural comparison of these five catalytic residues in WT-HaBLA and EaBLA, only a small side-chain rearrangement of the catalytic residues in WT-HaBLA was found. The r.m.s.d. for side-chain atoms between the active sites of WT-HaBLA and EaBLA was <1.4 Å (Fig. 3 ▶). The active site was positively charged owing to the existence of Lys68, His113, Arg149 and Lys316 even in the negatively charged circumstances of the overall structure, as mentioned above (Fig. 2 ▶).

In the vicinity of the active site of HaBLA, we also found a hydrophobic cluster composed of five hydrophobic and aromatic residues: Leu120, Phe121, Tyr290, Ile293 and Leu294 (Fig. 3 ▶). This number of residues is larger than that in EaBLA (Leu120, Ile292 and Leu293). The side chains of Leu120 and Ile293 of HaBLA form van der Waals contacts (within 4 Å distance) with the aromatic ring of Tyr151 in the active site, as observed in EaBLA.

### 3.3. Ca^2+^ binding observed in NQ-HaBLA without soaking   

The structural analysis of NQ-HaBLA shows that there are up to 11 locations for Ca^2+^ (Ca1–Ca11) in each asymmetric unit (Supplementary Table S1). The site for Ca1 consists of Asp56 and Glu170, the site for Ca2 consists of Asp58 and Asp128, the site for Ca3 consists of Asp85, Asp87 and Asp187, the site for Ca4 consists of Asp124, the site for Ca5 consists of Asp199, the sites for Ca6 (in chain *A*), Ca7 (in chain *B*) and Ca8 (in chain *C*) consist of Asp219 and Asp220 and the sites for Ca9 (in chain *A*), Ca10 (in chain *B*) and Ca11 (in chain *C*) consist of Asp291, Glu295 and Glu352. These 11 binding sites for Ca^2+^ were integrated into seven sites per molecule for NQ-HaBLA (Table 2 ▶).

The binding sites for Na^+^ were not identified in any of the measured HaBLA crystals because of its similar electron density to the O atom in water. In addition, the binding sites for Mg^2+^ in the structure of WT-HaBLA were not identified because of the noisy electron-density map owing to the low resolution of the X-ray diffraction (2.9 Å).

### 3.4. Cs^+^-soaking experiments for exploration of the binding sites in NQ-HaBLA   

**Figure 4:**
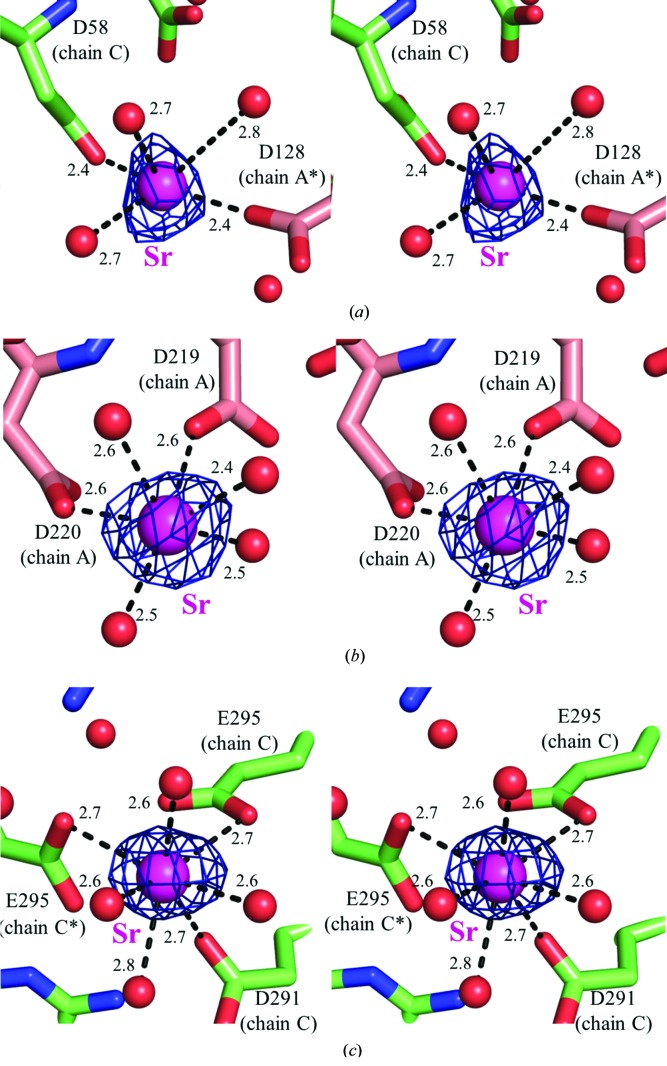

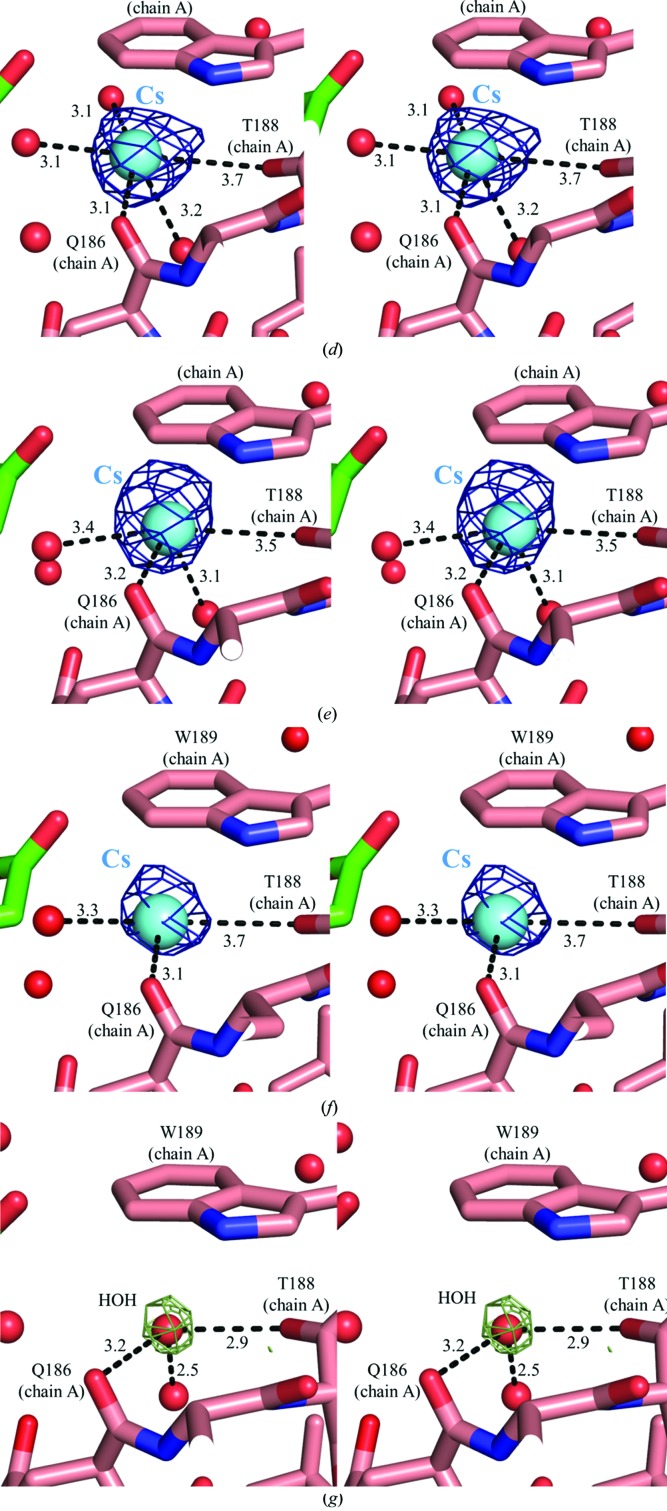
Relaxed stereoviews of the Sr^2+^- and Cs^+^-binding sites identified for NQ-HaBLA. (*a*), (*b*) and (*c*) show the site 2, site 6 and site 7 Sr^2+^-binding sites, respectively, observed in condition 2A (0 m*M* Ca^2+^/200 m*M* Sr^2+^). (*d*), (*e*) and (*f*) show the site 8 Cs^+^-binding site observed in condition 1A (0 m*M* Na^+^/100 m*M* Cs^+^), in condition 1B (75 m*M* Na^+^/25 m*M* Cs^+^) and in condition 1C (90 m*M* Na^+^/10 m*M* Cs^+^), respectively. (*g*) shows site 8 in the crystal structure without soaking (100 m*M* Na^+^/0 m*M* Cs^+^) as a control. The colours of chains *A*, *B* and *C* and of the metal ions are the same as those in Fig. 1 ▶. In (*a*) and (*c*), chains generated by a crystal symmetry operator are indicated by an asterisk after the chain name. The blue meshes in (*a*), (*b*), (*c*), (*d*), (*e*) and (*f*) show the anomalous difference Fourier map within a 5σ contour level. The green mesh in (*g*) shows a 2*F*_o_ − *F*_c_ *OMIT* map within a 2σ contour level.

The NQ-HaBLA crystals soaked into solutions containing Na^+^, Cs^+^ and Ca^2+^ [conditions 1A (0 m*M* Na^+^/100 m*M* Cs^+^), 1B (75 m*M* Na^+^/25 m*M* Cs^+^) and 1C (90 m*M* Na^+^/10 m*M* Cs^+^)] diffracted to resolutions of 2.0, 1.8 and 2.1 Å, respectively. All measured HaBLA crystals retained the same space group (*P*3_1_) and similar unit-cell parameters (Table 1 ▶). The peak heights of the anomalous difference Fourier maps of the observed Cs^+^ ions were higher than 7.5σ, as described below, which was higher than the background noise (<2σ) of other atoms. The structure of the Cs^+^-binding site in NQ-HaBLA is shown in Fig. 4 ▶.

Structural analysis of NQ-HaBLA in condition 1A (0 m*M* Na^+^/100 m*M* Cs^+^) showed ten locations for bound Ca^2+^ (Ca1–Ca10) and two locations for Cs^+^ (Cs1 and Cs2) in an asymmetric unit (Supplementary Table S1). The peak heights of the anomalous difference Fourier maps were 17.5σ for Cs1 and 8.0σ for Cs2. The binding sites of Ca^2+^ were integrated into five sites in one molecule of NQ-HaBLA, which were the same locations as the site 2, site 3, site 5, site 6 and site 7 Ca^2+^-binding sites in NQ-HaBLA without soaking (Table 2 ▶). Moreover, the binding sites of Cs^+^ ions were integrated into one site (site 8) per molecule of NQ-HaBLA.

Structural analysis of NQ-HaBLA in condition 1B (75 m*M* Na^+^/25 m*M* Cs^+^) showed ten locations for Ca^2+^ (Ca1–Ca10) and two locations for Cs^+^ (Cs1 and Cs2) in an asymmetric unit (Supplementary Table S1). The peak heights of the anomalous difference Fourier maps were 17.0σ for Cs1 and 7.5σ for Cs2. The binding sites of Ca^2+^ ions were integrated into six sites in one molecule of NQ-HaBLA, which were the same locations as the site 2, site 3, site 4, site 5, site 6 and site 7 Ca^2+^-binding sites in NQ-HaBLA without soaking (Table 2 ▶). Moreover, the binding sites of Cs^+^ ions were integrated into one site (site 8) per molecule of NQ-HaBLA. This Cs^+^-binding site was in the same location as the site 8 Cs^+^-binding site in condition 1A (Table 2 ▶).

Structural analysis of NQ-HaBLA in condition 1C (90 m*M* Na^+^/10 m*M* Cs^+^) showed ten locations for Ca^2+^ (Ca1–Ca10) and one location for Cs^+^ (Cs1) in an asymmetric unit (Supplementary Table S1). The peak height of the anomalous difference Fourier map was 9.0σ for Cs1. The binding sites of the Ca^2+^ ions were integrated into six sites in one molecule of NQ-HaBLA, which were the same locations as the site 2, site 3, site 4, site 5, site 6 and site 7 Ca^2+^-binding sites in NQ-HaBLA without soaking (Table 2 ▶). Moreover, the binding site of the Cs^+^ ion was in the same location as the site 8 Cs^+^-binding site in condition 1A (Table 2 ▶).

### 3.5. Structural characteristics of the Cs^+^-binding site observed in NQ-HaBLA   

As mentioned above, one Cs^+^-binding site (site 8) was discovered for conditions 1A (0 m*M* Na^+^/100 m*M* Cs^+^), 1B (75 m*M* Na^+^/20 m*M* Cs^+^) and 1C (90 m*M* Na^+^/10 m*M* Cs^+^) (Table 2 ▶).

Site 8 is located at the surface of a single HaBLA molecule and is comprised of two main-chain O atoms of Gln186 and Thr188 and the aromatic ring of Trp189 (Figs. 4 ▶ *d*, 4 ▶ *e* and 4 ▶ *f*). The aromatic ring of Trp189 interacts with Cs^+^ by a cation–π interaction. The distances between the identified Cs^+^ ion and the chelating main-chain O atoms in site 8 are slightly different in each molecule in the asymmetric unit and fall between 3.1 and 3.7 Å (Supplementary Table S1). In addition, the distance between the identified Cs^+^ ion and C atoms (C^δ2^, C^∊2^, C^∊3^, C^ζ2^, C^ζ3^ and C^η2^) of Trp189 are also slightly different in each molecule of the asymmetric unit and fall between 3.2 and 4.0 Å (Supplementary Table S1).

### 3.6. Sr^2+^-soaking experiments for exploration of the binding sites in NQ-HaBLA   

NQ-HaBLA crystals soaked in solutions containing Na^+^, Ca^2+^ and Sr^2+^ [conditions 2A (0 m*M* Ca^2+^/200 m*M* Sr^2+^) and 2B (100 m*M* Ca^2+^/100 m*M* Sr^2+^)] diffracted to resolutions of 1.9 and 2.8 Å, respectively. All measured HaBLA crystals retained the same space group (*P*3_1_) and similar unit-cell parameters (Table 1 ▶). The peak heights of the anomalous difference Fourier maps of the observed Sr^2+^ ions were >7.0σ, as described below, and were higher than the background noise or those of other atoms (<2σ). The structure of the Sr^2+^-binding site in NQ-HaBLA is shown in Fig. 4 ▶.

Structural analysis of NQ-HaBLA in condition 2A (0 m*M* Ca^2+^/200 m*M* Sr^2+^) showed eight locations for Sr^2+^ (Sr1–Sr8) in an asymmetric unit (Supplementary Table S1). The peak heights of the anomalous difference Fourier maps were 7.5σ for Sr1 and Sr2, 9σ for Sr5, 12σ for Sr4, 13σ for Sr3, 14σ for Sr7, 15σ for Sr8 and 16σ for Sr6. The binding sites of Sr^2+^ ions were integrated into three sites per molecule of NQ-HaBLA, which were the same locations as the site 2, site 6 and site 7 Ca^2+^-binding sites in NQ-HaBLA without soaking (Table 2 ▶). The Sr^2+^-binding structures at site 2, site 6 and site 7 in NQ-HaBLA in condition 2A are shown in Fig. 4 ▶.

Structural analysis of NQ-HaBLA in condition 2B (100 m*M* Ca^2+^/100 m*M* Sr^2+^) showed one location for Ca^2+^ (Ca1) and one location for Sr^2+^ (Sr1) in the asymmetric unit (Supplementary Table S1). The peak height of the anomalous difference Fourier map was 7.0σ for Sr1. The binding site for the Ca^2+^ ion was in the same location as the site 7 Ca^2+^-binding site in NQ-HaBLA without soaking (Table 2 ▶). Moreover, the binding site of the Sr^2+^ ion was in the same location as the site 6 Ca^2+^-binding site in NQ-HaBLA without soaking (Table 2 ▶).

From the comparison of the results from condition 2A and condition 2B, it was determined that site 6 binds Sr^2+^ rather than Ca^2+^ and that site 7 binds Ca^2+^ rather than Sr^2+^.

### 3.7. Structural characteristics of the Sr^2+^-binding sites in NQ-HaBLA   

As mentioned above, three Sr^2+^-binding sites (sites 2, 6 and 7) were discovered in conditions 2A (0 m*M* Ca^2+^/200 m*M* Sr^2+^) and 2B (100 m*M* Ca^2+^/100 m*M* Sr^2+^) (Table 2 ▶). Site 2 is located at the interface of a neighbouring chain in an asymmetric unit and was constructed of two O^δ1^ atoms from Asp58 and Asp128 (Fig. 4 ▶ *a*). The coordination number of the identified Sr^2+^ at site 2 is five. The distance between the identified Sr^2+^ and chelating O atoms in site 2 lies between 2.4 and 2.7 Å (Supplementary Table S1). Site 6 is located on the surface of a single HaBLA molecule and is constructed of the O^δ1^ atoms of Asp219 and Asp220 (Fig. 4 ▶ *b*). The observed coordination number of the identified Sr^2+^ at site 6 is distributed between four and six. The distance between the identified Sr^2+^ and chelating O atoms in site 6 lies between 2.3 and 2.9 Å (Supplementary Table S1). Site 7 is located at the interface with a neighbouring chain in the asymmetric unit and is constructed of the O^δ1^ atom of Asp291 and the O^∊1^ atoms of Glu295 and Glu352 (Fig. 4 ▶ *c*). The observed coordination number for the identified Sr^2+^ at site 7 is distributed between three and seven. The distance between the identified Sr^2+^ and chelating O atoms in site 7 is different in each molecule in the asymmetric unit and lies between 2.3 and 2.8 Å (Supplementary Table S1).

The average distances between Sr^2+^ and chelating O atoms in site 2, site 6 and site 7 of NQ-HaBLA are each 2.6 Å, which is very similar to those of known Sr^2+^-binding sites, including 2.6 Å in ribonuclease T1 (Deswarte *et al.*, 2001 ▶; PDB entry 1hyf), 2.6 Å in P-selectin (Somers *et al.*, 2000 ▶; PDB entry 1g1s), 2.5 Å in PLAP (Llinas *et al.*, 2006 ▶; PDB entry 2glq) and 2.6 Å in the oligomerization domain of rotavirus NSP4 (Bowman *et al.*, 2000 ▶; PDB entry 1g1j).

### 3.8. Salt dependency of HaBLA activity assessed by ITC   

**Figure 5:**
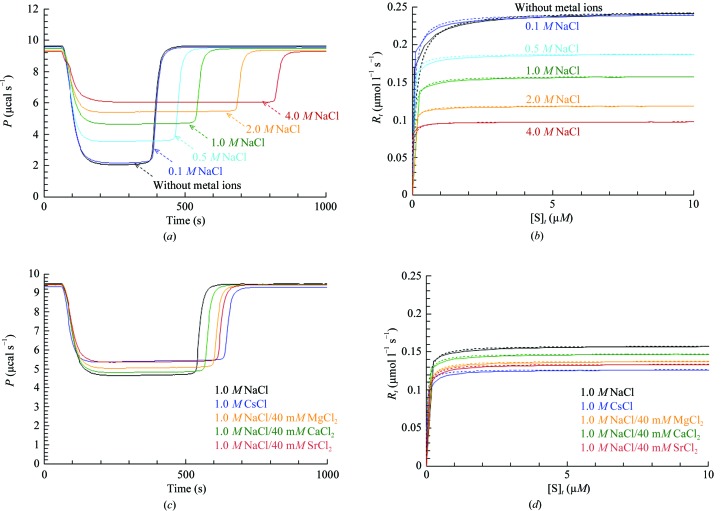
Calorimetric thermograms (figures on the left) and Michaelis–Menten plots (figures on the right) of penicillin G hydrolysis with HaBLA measured by ITC at 298.15 K. (*a*) and (*b*) show NaCl concentration dependency. (*c*) and (*d*) show metal ion dependency. In the Michaelis–Menten plots, theoretical data (dashed line) are fitted to experimental data (solid line).

**Figure 6:**
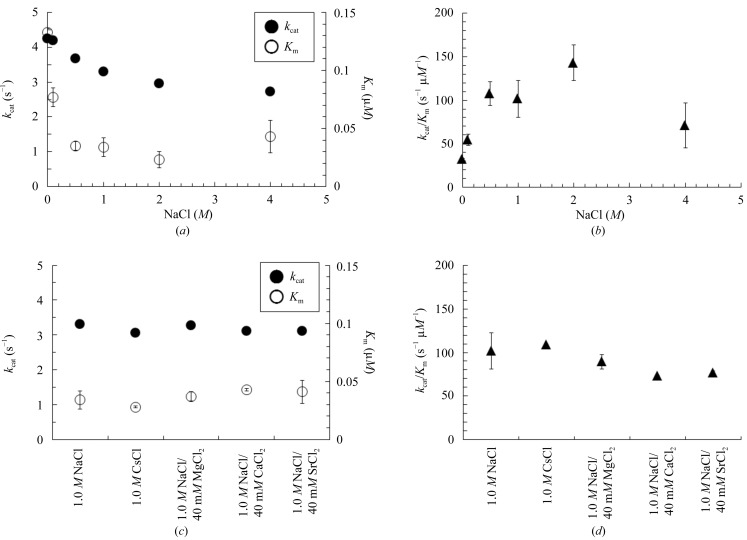
*k*_cat_, *K*_m_ and *k*_cat_/*K*_m_ estimated from the Michaelis–Menten plots in Fig. 5 ▶. (*a*) and (*b*) show NaCl concentration dependency. (*c*) and (*d*) show metal ion dependency.

The salt dependency of penicillin G hydrolysis by HaBLA was assessed by ITC. Exothermic calorimetric thermograms of the enzymatic reaction and Michaelis–Menten plots are shown in Fig. 5 ▶. The kinetic parameters (*k*_cat_, *K*_m_ and *k*_cat_/*K*_m_) estimated from Michaelis–Menten plots are summarized in Fig. 6 ▶ and Supplementary Table S2. The NaCl concentration dependency of the enzymatic activity of HaBLA was assessed for concentrations of NaCl from 0 to 4.0 *M*. Interestingly, HaBLA maintained enzymatic activity without metal ions (0 *M* NaCl), where the *k*_cat_, *K*_m_ and *k*_cat_/*K*_m_ values were 4.25 ± 0.04 s^−1^, 0.133 ± 0.003 µ*M* and 32.1 ± 0.9 s^−1^ µ*M* ^−1^, respectively. The value of *k*_cat_ decreased to 2.72 ± 0.05 s^−1^ on an increase in NaCl concentration to 4.0 *M*. The *K*_m_ value decreased to 0.023 ± 0.007 µ*M* on an increase in NaCl concentration to 2.0 *M* and increased again to 0.043 ± 0.014 µ*M* at 4.0 *M*. The maximum *k*_cat_/*K*_m_ value (142.8 ± 20.4 s^−1^ µ*M* ^−1^) was estimated to be at 2.0 *M* NaCl (Fig. 6*b* ▶ and Supplementary Table S2). Moreover, the enzymatic activities of HaBLA in the presence of 1.0 *M* NaCl and 1.0 *M* CsCl were compared in order to assess the monovalent metal-ion dependency of the enzymatic activity. The *k*_cat_, *K*_m_ and *k*_cat_/*K*_m_ values in the presence of 1.0 *M* CsCl were similar to those in 1.0 *M* NaCl, as shown in Figs. 6(*c*) and 6(*d*) ▶, and Supplementary Table S2 (the average values of *k*_cat_, *K*_m_ and *k*_cat_/*K*_m_ were 3.18 ± 0.05 s^−1^, 0.031 ± 0.005 µ*M* and 105.2 ± 11.0 s^−1^ µ*M* ^−1^, respectively).

The enzymatic activities of HaBLA in 1.0 *M* NaCl/40 m*M* MgCl_2_, 1.0 *M* NaCl/40 m*M* CaCl_2_ and 1.0 *M* NaCl/40 m*M* SrCl_2_ were also compared in order to assess the divalent metal-ion dependency of the enzymatic activity. The *k*_cat_, *K*_m_ and *k*_cat_/*K*_m_ values in the presence of 1.0 *M* NaCl/40 m*M* SrCl_2_ were similar to the values obtained in 1.0 *M* NaCl/40 m*M* MgCl_2_ and 1.0 *M* NaCl/40 m*M* CaCl_2_, as shown in Figs. 6(*c*) and 6(*d*) ▶, and Supplementary Table S2 (the average values of *k*_cat_, *K*_m_ and *k*_cat_/*K*_m_ are 3.17 ± 0.03 s^−1^, 0.040 ± 0.005 µ*M* and 79.6 ± 3.7 s^−1^ µ*M* ^−1^, respectively).

From these results, it is demonstrated that HaBLA maintains enzymatic activity even if Cs^+^ is present instead of Na^+^ and even if Sr^2+^ is present instead of Mg^2+^ and Ca^2+^.

## 4. Discussion   

HaBLA has an abundant content of surface acidic amino acids that have enabled adaptation to high salt concentrations. The crystal structure of HaBLA shows a unique active-site structure comprising the positively charged residues Lys68, His113, Arg149 and Lys316 and a hydrophobic cluster (Leu120, Phe121, Tyr290, Ile293 and Leu294), which helps to maintain the enzymatic function over a wide range of salt concentration (0–4.0 *M*) even in the negatively charged circumstances of the overall structure. Seven binding sites (site 1 to site 7) for divalent metal ions (Ca^2+^ and/or Sr^2+^) and one binding site (site 8) for Cs^+^ per HaBLA molecule were identified by X-ray diffraction studies including anomalous dispersion. Although metal ions belonging to the same group in the periodic table have similar chemical characteristics, at least two metal ion-binding sites exhibited specificity for different metal ions (*i.e.* site 6 selects Sr^2+^ rather than Ca^2+^ and site 8 selects Cs^+^ rather than Na^+^). To understand the mechanism of the metal-ion selectivity, the structural characteristics of the metal ion-binding sites are discussed.

### 4.1. Structural comparison of HaBLA with nonhalophilic BLAs and other halophilic proteins   

The structure of HaBLA is compared with the non­halophilic homologous BLAs EaBLA from *E. aerogenes* (PDB entry 1zkj, 1.55 Å resolution; Kim *et al.*, 2006 ▶), PfBLA from *Pseudomonas fluorescens* (PDB entry 2qz6, 2.26 Å resolution; Michaux *et al.*, 2008 ▶) and PaBLA from *P. aeruginosa* PAO1 (PDB entry 4nk3, 1.90 Å resolution; Blizzard *et al.*, 2014 ▶) (Table 3 ▶). These nonhalophilic homologous BLAs have moderate sequence identity (>48%) to HaBLA but a low r.m.s.d. on C^α^ atoms (<1.3 Å) (Table 3 ▶). While the solvent-accessible surface area (ASA) of HaBLA (14 093 Å^2^) is similar to those of nonhalophilic homologous BLAs (13 928–14 797 Å^2^), the acidic residue content [(Asp + Glu)/(Arg + Lys + His)] at the solvent-accessible surface of HaBLA (1.68) is about twice as large as those of the nonhalophilic BLAs (0.70–0.88). The number of negative charges [(Asp + Glu) − (Arg + Lys + His)] at the surface of HaBLA is calculated to be 21, which is also larger than those of nonhalophilic homologous BLAs (−5 to −12). From the ASA and the number of negative charges, the negative charge density at the surface of HaBLA is calculated to be 1.49 × 10^−3^ e Å^−2^, which is larger than those of nonhalophilic homologous BLAs (−0.34 × 10^−3^ to  −0.86 × 10^ 3^ e Å^−2^) (Table 3 ▶). In addition, no metal ion-binding sites of PfBLA and PaBLA were identified in previous studies (Michaux *et al.*, 2008 ▶; Blizzard *et al.*, 2014 ▶). Seven Zn^2+^-binding sites were identified in EaBLA (Kim *et al.*, 2006 ▶), but basic residues such as histidine contributed to the construction of these sites. From these results, it is suggested that the high density of negative charges on the surface of HaBLA may be suitable for the construction of binding sites for alkali-metal ions and alkaline-earth metal ions.

On the other hand, a high content of acidic residues in halophilic proteins may destabilize their structure and remove their enzymatic function under low salt concentrations owing to charge repulsion (although this would be effectively screened in >0.2 *M* salt). However, the results of the enzymatic assay indicated that HaBLA can hydrolyse penicillin G over a wide salt concentration range (*e.g.* 0–4.0 *M* NaCl). In order to understand the relationship between the structure and function of HaBLA, we compared the crystal structure of HaBLA with those of other halophilic proteins.

It is known that a dehydrofolate reductase (hv-DHFR; PDB entry 1vdr) from the extreme halophile *Haloferax volcanii* (Pieper *et al.*, 1998 ▶) has positive charges around the active site, whereas most of the remaining surface of hv-DHFR is negatively charged. These positive charges in hv-DHFR are expected to attract and bind negatively charged compounds to the active site (Pieper *et al.*, 1998 ▶). HaBLA also has positive charges surrounding the entrance to the active site (Fig. 2 ▶). Since penicillin G is a negatively charged substrate, the positive charges around the active site of HaBLA may be suitable for inducing penicillin G into the active site.

The hydrophobic cluster of HaBLA (Fig. 3 ▶), which may participate in a hydrophobic interaction with hydrophobic parts of a substrate (such as an aromatic ring of penicillin G), may also contribute to penicillin G hydrolysis. For example, the moderate halophilic alkaline phosphatase HaAP from *Halomonas* sp. 593 has a similar hydrophobic cluster at the entrance to the active site, which was expected to induce aromatic and aliphatic phosphoesters into the active site by hydrophobic interaction (Arai *et al.*, 2014 ▶). Even if electrostatic repulsion is caused between an anionic substrate such as penicillin G and the negatively charged surface of HaBLA under low salt concentrations, the hydrophobic interaction may assist in inducing penicillin G into the active site. When the salt concentration is high, the negatively charged surface of HaBLA will be effectively screened and the hydrophobic interaction will be improved by the salting-out effect.

Since the enzymatic activity of HaBLA was retained in solutions containing 1.0 *M* Cs^+^ or 1.0 *M* Sr^2+^ and at salt concentrations ranging from 0 *M* NaCl (without metal ions) to 4.0 *M* NaCl, the tertiary structure of HaBLA including the positive charges and the hydrophobic cluster is maintained under these solution conditions.

### 4.2. Sr^2+^ selectivity of HaBLA   

Three binding sites (sites 2, 6 and 7) for the recognition of Ca^2+^ or Sr^2+^ were successfully identified in NQ-HaBLA (Table 2 ▶). There are two mechanisms by which these metal-binding sites recognize different metal ions. One is the use of the crystal-packing interface, as seen in sites 2 and 7, and the other is the location of acidic residues, as observed in site 6. These divalent metal-binding sites have a relatively broad specificity for divalent alkaline-earth metal ions. Metal-ion recognition in sites 2, 6 and 7 is performed by the O^δ1^ atoms of Asp side chains and/or the O^∊1^ atoms of Glu side chains. An alternative rotamer orientation was observed for these side chains upon recognition of the different metal ions. The r.m.s.d. of these side chains among all observed HaBLA structures was <2.60 Å for site 2 (Asp58 and Asp128), <1.63 Å for site 6 (Asp219 and Asp220) and <2.65 Å for site 7 (Asp291, Glu295 and Glu352). Therefore, it can be concluded that side-chain structural adaptations to the ionic radius of the specific metal ions at sites 2, 6 and 7 appear to be responsible for enabling suitable coordination of the ligand O atoms.

Divalent metal-ion binding to HaBLA may also contribute to the association of HaBLA molecules. Several binding sites for divalent metal ions (such as sites 1, 2, 3 and 7) are located at the interface of HaBLA molecules; furthermore, HaBLA precipitated in crystallization mother liquor containing divalent metal ions at concentrations above 200 m*M*.

From these results, it is suggested that HaBLA has a broad spectrum of specificity for alkaline-earth metal ions and that the association of HaBLA molecules may be promoted by alkaline-earth metal ions. Sites 2 and 7 in monodisperse HaBLA may not bind Sr^2+^ in solution, since crystal packing is necessary to construct these sites. On the other hand, site 6 in monodisperse HaBLA may bind Sr^2+^ in solution, since the crystal-packing arrangement is not needed to construct this site. Therefore, structural information on site 6 might be useful in creating an artificial Sr^2+^-binding site.

### 4.3. Cs^+^ selectivity of HaBLA   

As mentioned previously, the site 8 Cs^+^-binding site is not located at the interface of HaBLA molecules but is constructed on the surface of a single HaBLA molecule (Fig. 4 ▶ and Table 2 ▶), indicating that monodisperse HaBLA itself can recognize Cs^+^. The Cs^+^ ion bound at site 8 was predominantly recognized by two main-chain O atoms and the aromatic ring of Trp189 (Fig. 4 ▶ and Table 2 ▶). Similar Cs^+^ recognition by an aromatic side chain has been seen in crystal structures of 3,4-dihydroxy-2-butanone 4-phosphate synthase (Liao *et al.*, 2001 ▶; PDB entry 1g57), the guanine nucleotide-binding protein G(I) (Kimple *et al.*, 2002 ▶; PDB entry 1kjy), subtilisin (Cianci *et al.*, 2010 ▶; PDB entry 2wuv) and the potassium inwardly rectifying channel (Inanobe *et al.*, 2011 ▶; PDB entry 3atf). The Cs^+^ selectivity of these proteins was not determined, since the location of Cs^+^ in these crystal structures was identified only in the presence of a high concentration of Cs^+^ and low (or zero) concentrations of other metal ions (*e.g.* 6 *M* Cs^+^ for 1g57, 1.4 *M* Cs^+^, 0.1 *M* Na^+^ and 1 m*M* Mg^2+^ for 1kjy, 1.5 *M* Cs^+^ for 2wuv and 200 m*M* Cs^+^ and 10 m*M* Ba^2+^ for 3atf).

On the other hand, we found that site 8 can recognize Cs^+^ selectively from the mother liquor even when it contained a ninefold greater concentration of Na^+^ over Cs^+^ (condition 1C; 90 m*M* Na^+^/10 m*M* Cs^+^), indicating that site 8 shows a greater affinity for Cs^+^ than Na^+^. Because the r.m.s.d. for the main-chain O atoms and the side chain of Trp189 at site 8 among all observed HaBLA structures is small (<0.18 Å), it can be concluded that the rigid conformation of the site 8 Cs^+^-binding site must be conserved in the presence of Na^+^, Mg^2+^, Ca^2+^, Sr^2+^ and/or Cs^+^, and this retention of the conformation is postulated to be responsible for the specificity for Cs^+^.

In conclusion, we succeeded in discovering a Cs^+^-selective binding site in the halophilic protein HaBLA. Future studies include the following. (i) Transplantation of the structure of the Cs^+^-selective binding site from HaBLA to other proteins: since it may be possible to create an artificial metal ion-binding site using this structural information (Kuroki *et al.*, 1989 ▶; Kuroki & Yutani, 1998 ▶), the design of artificial protein absorbents for Cs^+^ might be possible by using the structural information on the Cs^+^-selective binding site of HaBLA. (ii) Improvement of the Cs^+^ affinity and/or selectivity of HaBLA: if the number of chelating O atoms with suitable ionic bond distances to Cs^+^ (3.02–3.30 Å; Haynes *et al.*, 2013 ▶; Jia, 1991 ▶; Shannon, 1976 ▶) is increased by a mutation, the Cs^+^ affinity and/or the selectivity of HaBLA may be further improved. (iii) A search for proteins having a Cs^+^-selective binding site: if proteins having a structure similar to the Cs^+^-selective binding site of HaBLA are identified in the PDB, these proteins might be useful as a scaffold to create a protein absorbent for Cs^+^.

## Supplementary Material

PDB reference: β-lactamase, wild type, 3wrt

PDB reference: N288Q/N321Q mutant, 3wrz

PDB reference: 3ws0

PDB reference: 3ws1

PDB reference: 3ws2

PDB reference: 3ws4

PDB reference: 3ws5

Supporting Information.. DOI: 10.1107/S1399004714027734/mn5080sup1.pdf

## Figures and Tables

**Table 1:** X-ray data-collection and refinement statistics for HaBLAs using a wavelength of 1.000 Values in parentheses are for the highest resolution shell.

		NQ-HaBLA					
	WT-HaBLA	Without soaking	Condition 1A	Condition 1B	Condition 1C	Condition 2A	Condition 2B
Metal ion in the mother liquor	0.2*M* Na^+^, 0.2*M* Mg^2+^	0.1*M* Na^+^, 0.2*M* Ca^2+^	0.1*M* Cs^+^, 0.2*M* Ca^2+^	25m*M* Cs^+^, 75m*M* Na^+^, 0.2*M* Ca^2+^	10m*M* Cs^+^, 90m*M* Na^+^, 0.2*M* Ca^2+^	0.1*M* Na^+^, 0.2*M* Sr^2+^	0.1*M* Na^+^, 0.1*M* Sr^2+^, 0.1*M* Ca^2+^
Beamline	NE3A, PF	NW12A, PF-AR	BL17A, PF	BL-7, SAGA-LS	BL-7, SAGA-LS	NW12A, PF-AR	NW12A, PF-AR
Space group	*P*3_1_	*P*3_1_	*P*3_1_	*P*3_1_	*P*3_1_	*P*3_1_	*P*3_1_
Unit-cell parameters (, )	*a* = 116.2, *b* = 116.2, *c* = 68.0, = 90, = 90, = 120	*a* = 114.9, *b* = 114.9, *c* = 67.6, = 90, = 90, = 120	*a* = 115.4, *b* = 115.4, *c* = 67.7, = 90, = 90, = 120	*a* = 115.1, *b* = 115.1, *c* = 67.3, = 90, = 90, = 120	*a* = 115.3, *b* = 115.3, *c* = 67.5, = 90, = 90, = 120	*a* = 115.0, *b* = 115.0, *c* = 67.8, = 90, = 90, = 120	*a* = 114.3, *b* = 114.3, *c* = 67.0, = 90, = 90, = 120
Resolution ()	2.90 (2.982.90)	1.80 (1.851.80)	2.00 (2.052.00)	1.80 (1.851.80)	2.10 (2.152.10)	1.90 (1.951.90)	2.80 (2.872.80)
No. of measured reflections	119812	500141	245651	529901	185618	199390	64156
No. of unique reflections	20547 (2005)	92589 (4610)	66954 (3384)	184880 (29383)	109679 (17296)	77242 (3875)	21928 (1114)
Multiplicity	5.8 (5.8)	5.4 (4.8)	3.7 (4.0)	5.7 (5.6)	3.2 (2.4)	2.6 (2.4)	2.9 (2.4)
*R*_merge_ † (%)	9.6 (35.2)	8.3 (26.3)	8.1 (31.2)	4.1 (30.8)	7.9 (40.9)	6.1 (38.7)	9.5 (39.8)
Completeness (%)	99.9 (99.8)	99.9 (99.4)	97.7 (99.9)	99.2 (98.0)	93.7 (91.5)	97.7 (97.2)	90.6 (92.4)
*I*/(*I*)	5.5 (5.5)	23.1 (15.5)	15.3 (12.4)	18.4 (3.8)	8.7 (1.9)	18.7 (5.6)	19.5 (8.1)
*R* factor‡ (%)	17.1 (29.3)	17.2 (15.3)	19.0 (21.9)	18.5 (20.4)	14.4 (12.5)	18.2 (15.8)	17.6 (18.3)
*R*_free_ (%)	22.5 (35.1)	22.1 (19.8)	22.4 (26.0)	21.1 (22.8)	17.4 (17.0)	21.6 (22.1)	20.7 (27.4)
Mean *B* value‡ (^2^)	41.7	20.2	30.4	31.4	28.9	31.9	44.0
Metal ions in asymmetric unit		11 Ca^2+^	2 Cs^+^, 10 Ca^2+^	2 Cs^+^, 10 Ca^2+^	1 Cs^+^, 10 Ca^2+^	8 Sr^2+^	1 Sr^2+^, 1 Ca^2+^
Stereochemistry§							
R.m.s.d., bonds ()	0.012	0.012	0.009	0.011	0.008	0.015	0.014
R.m.s.d., angles ()	1.551	1.789	1.417	1.573	1.412	1.603	1.585
Ramachandran analysis¶ (%)							
Favoured regions	88.8	90.5	91.2	90.5	91.3	91.1	89.3
Allowed	10.6	9.1	8.5	8.9	8.4	8.6	10.3
Disallowed	0.6	0.4	0.3	0.6	0.3	0.3	0.4
PDB entry	3wrt	3wrz	3ws0	3ws1	3ws2	3ws4	3ws5

† *R*_merge_ = 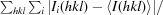 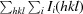.

‡ *R* factor and *R*_free_ = 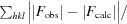 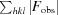, where the free reflections (5% of the total used) were held aside for the calculation of *R*_free_ throughout the refinement.

§ Deviation from ideal values.

¶ Ramachandran analysis was carried out using *RAMPAGE* (Lovell *et al.*, 2003 ▶)

**Table 2:** Metal ion-binding sites of NQ-HaBLA classified based on ligand residues Chains generated by a crystal symmetry operator are indicated by an asterisk after the chain name. Square brackets indicate the chains involving the residues of the metal ion-binding sites. Parentheses indicate the locations of the metal ion-binding site. For example, (*A*, *C*) means that the metal ion-binding sites is located at the monomer surfaces of each chain *A* and chain *C* and (*A*/*B*) means that the metal ion-binding site is located at the interface between chain *A* and chain *B*.

			Bound metal ions					
	Chelating atoms		Without soaking	Condition 1A	Condition 1B	Condition 1C	Condition 2A	Condition 2B
Metal ion-binding sites	Residue	Atom	200m*M* Na^+^, 200m*M* Ca^2+^	100m*M* Cs^+^, 200m*M* Ca^2+^	25m*M* Cs^+^, 75m*M* Na^+^, 200m*M* Ca^2+^	10m*M* Cs^+^, 90m*M* Na^+^, 200m*M* Ca^2+^	100m*M* Na^+^, 200m*M* Sr^2+^	100 m*M* Na^+^, 100m*M* Sr^2+^, 100m*M* Ca^2+^
Site 1	Asp56 [*A*]	O^1 ^	Ca^2+^ (*A*/*C*)					
	Glu170 [*C*]	O^1^						
	HOH 3	O						
Site 2	Asp58 [*C*]	O^1^	Ca^2+^ (*C*/A*)	Ca^2+^ (*C*/A*)	Ca^2+^ (*C*/A*)	Ca^2+^ (*C*/A*)	Sr^2+^ (*C*/A*)	
	Asp128 [A*]	O^1^						
	HOH 35	O						
Site 3	Asp85 [*A*]	O^1 ^	Ca^2+^ (*A*/*B*)	Ca^2+^ (*A*/*B*)	Ca^2+^ (*A*/*B*)	Ca^2+^ (*A*/*B*)		
	Asp87 [*A*]	O^1 ^						
	Asp187 [*B*]	O^1^						
	HOH 12	O						
Site 4	Asp124 [*A*]	O^1^	Ca^2+^ (*A*)		Ca^2+^ (*A*)	Ca^2+^ (*A*)		
	HOH 35	O						
Site 5	Asp199 [*B*]	O^1^	Ca^2+^ (*B*)	Ca^2+^ (*A*, *B*)	Ca^2+^ (*B*)	Ca^2+^ (*B*)		
	HOH 23	O						
Site 6	Asp219 [*A*, *B*, *C*]	O^1^	Ca^2+^ (*A*, *B*, *C*)	Ca^2+^ (*A*, *B*, *C*)	Ca^2+^ (*A*, *B*, *C*)	Ca^2+^ (*A*, *B*, *C*)	Sr^2+^ (*A*, *B*, *C*)	Sr^2+^ (*A*)
	Asp220 [*A*, *B*, *C*]	O^1 ^						
	HOH 15	O						
Site 7	Asp291 [A*, *B*, C*]	O^1 ^	Ca^2+^ (*A*/A*, *B*/B*, *C*/C*)	Ca^2+^ (*A*/A*, *B*/B*, *C*/C*)	Ca^2+^ (*A*/A*, *B*/B*, *C*/C*)	Ca^2+^ (*A*/A*, *B*/B*, *C*/C*)	Sr^2+^ (*A*/A*, *B*/B*, *C*/C*)	Ca^2+^ (*B*/B*)
	Glu295 [A*, *B*, C*]	O^1^						
	Glu352 [*A*, B*, *C*]	O^1^						
	HOH 14	O						
Site 8	Gln186 [*A*, *C*]	O		Cs^+^ (*A*, *C*)	Cs^+^ (*A*, *C*)	Cs^+^ (*A*)		
	Thr188 [*A*, *C*]	O						
	Trp189† [*A*, *C*]	C^2^, C^2^, C^3^, C^2^, C^3^, C^2^						
	HOH 01	O						

† The aromatic ring of Trp189 interacts with Cs^+^ by a cation interaction.

**Table 3:** Structural comparison between HaBLA and nonhalophilic BLAs

-Lactamase	NQ-HaBLA (this work)	EaBLA (PDB entry 1zkj)	PfBLA (PDB entry 2qz6)	PaBLA (PDB entry 4nk3)
Biological source	*Chromohalobacter* sp. 560	*E. aerogenes* (nonhalophilic)	*P. fluorescens* (nonhalophilic)	*P. aeruginosa* PAO1 (nonhalophilic)
Resolution ()	2.901.80	1.55	2.26	1.90
No. of identified metal ion-binding sites	8	7	0	0
Sequence identity (%)	100	48	48	48
R.m.s.d. on C atoms ()		1.3	1.2	1.2
Volume† (^3^)	55883	54351	54835	57986
Acessible surface areas				
ASA (^2^)	14093	13928	13941	14797
ASA of nonpolar residues (^2^)	4880	4831	4656	4658
ASA of polar residues (^2^)	9213	9097	9285	10140
Amino-acid composition				
Nonpolar/polar residues‡	1.17 (198/169)	1.23 (198/161)	1.11 (188/170)	1.19 (204/171)
Acidic residues (Asp + Glu)	32 + 25	12 + 17	15 + 15	23 + 15
Basic residues (Arg + Lys + His)	18 + 9 + 5	13 + 18 + 10	5 + 23 + 9	24 + 16 + 6
(Asp + Glu)/(Arg + Lys + His)	1.78	0.71	0.81	0.83
Solvent-accessible surface residues (ASA > 0^2^)				
Nonpolar/polar residues‡	1.03 (162/157)	1.07 (161/150)	1.03 (156/152)	1.03 (163/159)
Acidic residues (Asp + Glu)	28 + 24	12 + 16	13 + 13	23 + 14
Basic residues (Arg + Lys + His)	17 + 9 + 5	13 + 17 + 10	5 + 22 + 9	24 + 12 + 6
(Asp + Glu)/(Arg + Lys + His)	1.68	0.70	0.72	0.88
Density of negative charge (e^2^)	1.49 10^3^	0.86 10^3^	0.72 10^3^	0.34 10^3^

† The web-based program 3*V* was used for this calculation (Voss Gerstein, 2010 ▶).

‡ Nonpolar residues are Gly, Ala, Val, Leu, Ile, Pro, Phe, Met and Trp. Polar residues are Asp, Glu, Arg, Lys, His, Asn, Gln, Ser, Thr, Tyr and Cys.
