# Supplementary material for: Structure of a highly acidic β-lactamase from the moderate halophile Chromohalobacter sp. 560 and the discovery of a Cs+-selective binding site
Source: Acta Crystallogr D Biol Crystallogr. 2015 Feb 26;71(Pt 3):541–54. doi: 10.1107/S1399004714027734 (PMC4356365; doi:10.1107/S1399004714027734)
Supplement: Supplementary file 1 [file d-71-00541-sup1.pdf]

BIOLOGICAL
CRYSTALLOGRAPHY

Volume 71 (2015)

Supporting information for article:

Structure of highly acidic β-lactamase from moderate halophile *Chromohalobacter* sp. 560 and the discovery of a Cs^{+} selective binding site

Shigeki Arai, Yasushi Yonezawa, Nobuo Okazaki, Fumiko Matsumoto, Chie Shibazaki, Rumi Shimizu, Mitsugu Yamada, Motoyasu Adachi, Taro Tamada, Masahide Kawamoto, Hiroko Tokunaga, Matsujiro Ishibashi, Michael Blaber, Masao Tokunaga and Ryota Kuroki

Table S1 Metal ions observed in NQ-HaBLA crystals.

Crystal	Site	Metal	No.	Distance (Å)	Ligands			
					Atom	Residue	No.	Chain
Without soaking	Site-1	Ca	1	2.6	Oδ1	Asp	56	A
Na^+ / Cs^+ = 100 mM/0 mM				2.6	Oε1	Glu	170	C
Ca^{2+} / Sr^{2+} = 200 mM/0 mM				2.5	O	HOH	543	S
				2.6	O	HOH	689	S
				2.4	O	HOH	690	S
	Site-2	Ca	2	2.3	Oδ1	Asp	58	C
				2.4	Oδ1	Asp	128	A*
				2.3	O	HOH	29	S
				2.5	O	HOH	33	S
				2.4	O	HOH	314	S
				2.6	O	HOH	402	S*
				2.3	O	HOH	608	S
	Site-3	Ca	3	2.4	Oδ1	Asp	85	A
				2.5	Oδ1	Asp	87	A
				2.3	Oδ1	Asp	187	B
				2.4	O	HOH	109	S
				2.4	O	HOH	110	S
	Site-4	Ca	4	2.1	Oδ1	Asp	124	A
				2.4	O	HOH	46	S
				2.7	O	HOH	234	S
				2.7	O	HOH	274	S
				2.4	O	HOH	364	S
				2.6	O	HOH	622	S
	Site-5	Ca	5	2.5	Oδ1	Asp	199	B
				2.4	O	HOH	211	S
				2.6	O	HOH	515	S
				2.8	O	HOH	747	S
	Site-6	Ca	6	2.5	Oδ1	Asp	219	A
				2.4	Oδ1	Asp	220	A
				2.5	O	HOH	64	S
				2.4	O	HOH	113	S
				2.4	O	HOH	276	S
				2.3	O	HOH	317	S
				2.4	O	HOH	318	S
		Ca	7	2.6	Oδ1	Asp	219	B
				2.4	Oδ1	Asp	220	B
				2.6	O	HOH	65	S
				2.2	O	HOH	114	S
				2.4	O	HOH	115	S
				2.3	O	HOH	116	S
				2.5	O	HOH	294	S

		Ca	8	2.7	Oδ1	Asp	219	C
				2.5	O	HOH	135	S
				2.1	O	HOH	330	S
				2.4	O	HOH	702	S
				2.4	O	HOH	729	S
	Site-7	Ca	9	2.3	Oδ1	Asp	291	A*
				2.3	Oε1	Glu	295	A*
				2.5	Oε1	Glu	352	A
				2.1	O	HOH	93	S*
				2.4	O	HOH	94	S
				2.5	O	HOH	107	S
				2.8	O	HOH	108	S
		Ca	10	2.4	Oδ1	Asp	291	B
				2.3	Oε1	Glu	295	B
				2.5	Oε1	Glu	352	B*
				2.3	O	HOH	78	S
				2.4	O	HOH	104	S
				2.2	O	HOH	284	S*
		Ca	11	2.6	Oδ1	Asp	291	C*
				2.4	Oε1	Glu	295	C*
				2.6	Oε1	Glu	352	C
				2.3	O	HOH	91	S*
				2.2	O	HOH	92	S*
				2.4	O	HOH	118	S
Condition-1A	Site-2	Ca	1	2.2	Oδ1	Asp	58	C
Na^+ / Cs^+ = 0 mM/100 mM				2.3	Oδ1	Asp	128	A*
Ca^{2+} / Sr^{2+} = 200 mM/0 mM				2.2	O	HOH	30	S
				2.1	O	HOH	456	S*
				2.4	O	HOH	526	S
	Site-3	Ca	2	2.5	Oδ1	Asp	85	A
				2.5	Oδ1	Asp	87	A
				2.3	Oδ1	Asp	187	B
				2.1	O	HOH	461	S
				2.3	O	HOH	462	S
	Site-5	Ca	3	2.4	Oδ1	Asp	199	A
				2.6	O	HOH	378	S
				2.4	O	HOH	538	S
		Ca	4	2.4	Oδ1	Asp	199	B
				2.5	O	HOH	399	S
				2.4	O	HOH	491	S
	Site-6	Ca	5	2.6	Oδ1	Asp	219	A
				2.5	Oδ1	Asp	220	A
				2.5	O	HOH	57	S
				2.5	O	HOH	188	S
				2.2	O	HOH	471	S

		Ca	6	2.6	Oδ1	Asp	219	B
				2.6	Oδ1	Asp	220	B
				2.7	O	HOH	58	S
				2.3	O	HOH	201	S
				2.5	O	HOH	527	S
		Ca	7	2.7	Oδ1	Asp	219	C
				2.6	Oδ1	Asp	220	C
				2.7	O	HOH	103	S
				2.8	O	HOH	335	S
				2.4	O	HOH	566	S
	Site-7	Ca	8	2.4	Oδ1	Asp	291	A*
				2.6	Oε1	Glu	295	A*
				2.5	Oε1	Glu	352	A
				2.1	O	HOH	80	S*
				2.4	O	HOH	81	S
				2.4	O	HOH	92	S
		Ca	9	2.4	Oδ1	Asp	291	B
				2.5	Oε1	Glu	295	B
				2.6	Oε1	Glu	352	B*
				2.6	O	HOH	90	S
				2.3	O	HOH	195	S*
				2.3	O	HOH	473	S
		Ca	10	2.6	Oε1	Glu	295	C*
				2.5	Oε1	Glu	352	C
				2.0	O	HOH	79	S*
				2.3	O	HOH	509	S*
				2.6	O	HOH	530	S
				2.5	O	HOH	549	S*
	Site-8	Cs	1	3.1	O	Gln	186	A
				3.7	O	Thr	188	A
				3.9	Cδ2	Trp	189	A
				3.7	Cε2	Trp	189	A
				3.8	Cε3	Trp	189	A
				3.5	Cζ2	Trp	189	A
				3.5	Cζ3	Trp	189	A
				3.3	Cη2	Trp	189	A
		Cs	2	3.3	O	Gln	186	C
				3.3	O	Thr	188	C
				3.7	Cδ2	Trp	189	C
				3.6	Cε2	Trp	189	C
				3.3	Cε3	Trp	189	C
				3.5	Cζ2	Trp	189	C
				3.4	Cζ3	Trp	189	C
				3.4	Cη2	Trp	189	C
Condition-1B	Site-2	Ca	1	2.4	Oδ1	Asp	58	C
Na^+ / Cs^+ = 75 mM25 mM				2.2	Oδ1	Asp	128	A*

Ca^{2+} / Sr^{2+} = 200 mM/0 mM				2.4	O	HOH	110	S
				2.5	O	HOH	441	S
				1.7	O	HOH	587	S
				2.2	O	HOH	588	S
	Site-3	Ca	2	2.5	Oδ1	Asp	85	A
				2.7	Oδ1	Asp	87	A
				2.4	Oδ1	Asp	187	B
				2.4	O	HOH	92	S
	Site-4	Ca	3	2.1	Oδ1	Asp	124	A
				2.4	O	HOH	233	S
				2.6	O	HOH	443	S
				2.6	O	HOH	572	S*
				2.2	O	HOH	592	S
	Site-5	Ca	4	2.6	Oδ1	Asp	199	B
				2.4	O	HOH	327	S
				2.4	O	HOH	350	S
				2.4	O	HOH	482	S
	Site-6	Ca	5	2.5	Oδ1	Asp	219	A
				2.4	Oδ1	Asp	220	A
				2.5	O	HOH	57	S
				2.5	O	HOH	85	S
				2.5	O	HOH	150	S
				2.4	O	HOH	187	S
				2.4	O	HOH	210	S
		Ca	6	2.4	Oδ1	Asp	219	B
				2.4	Oδ1	Asp	220	B
				2.5	O	HOH	322	S
				2.4	O	HOH	394	S
		Ca	7	2.6	Oδ1	Asp	219	C
				2.7	Oδ1	Asp	220	C
				2.8	O	HOH	171	S
				2.3	O	HOH	329	S
				2.5	O	HOH	457	S
	Site-7	Ca	8	2.3	Oδ1	Asp	291	A*
				2.6	Oε1	Glu	295	A*
				2.5	Oε1	Glu	352	A
				2.1	O	HOH	114	S
				2.4	O	HOH	123	S
				2.3	O	HOH	139	S
		Ca	9	2.4	Oδ1	Asp	291	B
				2.7	Oε1	Glu	295	B
				2.5	Oε1	Glu	352	B*
				2.2	O	HOH	221	S
				2.6	O	HOH	374	S
		Ca	10	2.7	Oδ1	Asp	291	C*
				2.4	Oε1	Glu	295	C*

				2.4	Oε1	Glu	352	C
				2.5	O	HOH	337	S
				1.6	O	HOH	591	S
	Site-8	Cs	1	3.2	O	Gln	186	A
				3.5	O	Thr	188	A
				4.0	Cδ2	Trp	189	A
				3.8	Cε2	Trp	189	A
				3.9	Cε3	Trp	189	A
				3.4	Cζ2	Trp	189	A
				3.9	Cζ3	Trp	189	A
				3.2	Cη2	Trp	189	A
				3.4	O	HOH	68	S
				3.7	O	HOH	571	S
		Cs	2	3.4	O	Gln	186	C
				3.2	O	Thr	188	C
				3.8	Cδ2	Trp	189	C
				3.9	Cε2	Trp	189	C
				3.6	Cε3	Trp	189	C
				3.7	Cζ2	Trp	189	C
				3.3	Cζ3	Trp	189	C
				3.4	Cη2	Trp	189	C
Condition-1C	Site-2	Ca	1	2.2	Oδ1	Asp	58	C
Na^+ / Cs^+ = 90 mM/10 mM				2.4	Oδ1	Asp	128	A*
Ca^{2+} / Sr^{2+} = 200 mM/0 mM				2.6	O	HOH	110	S
				2.7	O	HOH	389	S
				2.4	O	HOH	493	S
	Site-3	Ca	2	2.6	Oδ1	Asp	85	A
				2.4	Oδ1	Asp	87	A
				2.5	Oδ1	Asp	187	B
				2.5	O	HOH	92	S
				2.7	O	HOH	531	S
	Site-4	Ca	3	2.2	Oδ1	Asp	124	A
				2.6	O	HOH	229	S
				2.4	O	HOH	391	S
				2.7	O	HOH	485	S*
	Site-5	Ca	4	2.3	Oδ1	Asp	199	B
				2.2	O	HOH	311	S
				2.3	O	HOH	331	S
	Site-6	Ca	5	2.6	Oδ1	Asp	219	A
				2.5	Oδ1	Asp	220	A
				2.8	O	HOH	57	S
				2.4	O	HOH	85	S
				2.2	O	HOH	150	S
				2.7	O	HOH	186	S
				2.3	O	HOH	208	S
		Ca	6	2.6	Oδ1	Asp	219	B

				2.3	Oδ1	Asp	220	B
				2.4	O	HOH	358	S
		Ca	7	2.6	Oδ1	Asp	219	C
				2.6	Oδ1	Asp	220	C
				2.7	O	HOH	170	S
				2.5	O	HOH	312	S
				2.8	O	HOH	404	S
	Site-7	Ca	8	2.4	Oδ1	Asp	291	A*
				2.4	Oε1	Glu	295	A*
				2.5	Oε1	Glu	352	A
				2.2	O	HOH	114	S
				2.4	O	HOH	123	S
				2.4	O	HOH	139	S
		Ca	9	2.4	Oδ1	Asp	291	B
				2.6	Oε1	Glu	295	B
				2.6	Oε1	Glu	352	B*
				2.2	O	HOH	218	S
		Ca	10	2.4	Oε1	Glu	295	C*
				2.5	Oε1	Glu	352	C
				2.6	O	HOH	319	S
				1.9	O	HOH	496	S
	Site-8	Cs	1	3.6	O	Gln	186	A
				3.7	O	Thr	188	A
				4.0	Cδ2	Trp	189	A
				3.9	Cε2	Trp	189	A
				3.7	Cε3	Trp	189	A
				3.6	Cζ2	Trp	189	A
				3.4	Cζ3	Trp	189	A
				3.4	Cη2	Trp	189	A
Condition-2A	Site-2	Sr	1	2.4	Oδ1	Asp	58	B
Na^+ / Cs^+ = 100 mM/0 mM				2.5	O	HOH	82	S
Ca^{2+} / Sr^{2+} = 0 mM/200 mM				2.7	O	HOH	438	S
				2.5	O	HOH	454	S
		Sr	2	2.4	Oδ1	Asp	58	C
				2.4	Oδ1	Asp	128	A*
				2.7	O	HOH	257	S
				2.7	O	HOH	386	S*
				2.7	O	HOH	444	S
	Site-6	Sr	3	2.6	Oδ1	Asp	219	A
				2.6	Oδ1	Asp	220	A
				2.6	O	HOH	48	S
				2.4	O	HOH	182	S
				2.5	O	HOH	211	S
				2.5	O	HOH	502	S
		Sr	4	2.6	Oδ1	Asp	219	B
				2.6	Oδ1	Asp	220	B

				2.6	O	HOH	49	S
				2.6	O	HOH	84	S
				2.6	O	HOH	194	S
				2.7	O	HOH	439	S
		Sr	5	2.7	Oδ1	Asp	219	C
				2.6	Oδ1	Asp	220	C
				2.5	O	HOH	96	S
				2.7	O	HOH	369	S
				2.8	O	HOH	310	S
	Site-7	Sr	6	2.6	Oδ1	Asp	291	A*
				2.7	Oε1	Glu	295	A*
				2.6	Oε1	Glu	352	A
				2.5	O	HOH	72	S*
				2.6	O	HOH	73	S
				2.5	O	HOH	508	S
		Sr	7	2.6	Oδ1	Asp	291	B
				2.7	Oε1	Glu	295	B
				2.6	Oε1	Glu	352	B*
				2.7	O	HOH	404	S
				2.3	O	HOH	449	S
		Sr	8	2.7	Oδ1	Asp	291	C*
				2.7	Oε1	Glu	295	C*
				2.7	Oε1	Glu	352	C
				2.8	O	HOH	70	S*
				2.6	O	HOH	71	S*
				2.6	O	HOH	85	S
				2.6	O	HOH	86	S
Condition-2B	Site-6	Sr	1	2.9	Oδ1	Asp	219	A
Na^+ / Cs^+ = 100 mM/0 mM				2.9	Oδ1	Asp	220	A
Ca^{2+} / Sr^{2+} = 100 mM/100 mM				2.3	O	HOH	15	S
				2.3	O	HOH	90	S
	Site-7	Ca	1	2.5	Oδ1	Asp	291	B
				2.7	Oε1	Glu	295	B
				2.5	Oε1	Glu	352	B*

* The neighboring chain of an asymmetric unit that is generated by a symmetric operation.

Table S2 Kinetic parameters (k_{cat}, K_M and k_{cat} / K_M) of penicillin G hydrolysis with HaBLA obtained by ITC at 25 °C.

**Metal Chloride**	k_{cat} **(S^{-1})**	K_M **(μM)**	k_{cat} / K_M **(S^{-1} μM^{-1})**
none	4.25 ± 0.04	0.133 ± 0.003	32.1 ± 0.9
0.1 M NaCl	4.19 ± 0.06	0.077 ± 0.008	54.8 ± 6.4
0.5 M NaCl	3.67 ± 0.03	0.035 ± 0.004	107.7 ± 13.7
1.0 M NaCl	3.30 ± 0.06	0.034 ± 0.008	101.5 ± 20.8
2.0 M NaCl	2.96 ± 0.06	0.023 ± 0.007	142.8 ± 20.4
4.0 M NaCl	2.72 ± 0.05	0.043 ± 0.014	71.0 ± 25.6
1.0 M NaCl	3.30 ± 0.06	0.034 ± 0.008	101.5 ± 20.8
1.0 M CsCl	3.06 ± 0.03	0.028 ± 0.001	108.9 ± 1.1
1.0 M NaCl / 40 mM $MgCl_2$	3.27 ± 0.01	0.037 ± 0.004	89.3 ± 8.0
1.0 M NaCl / 40 mM $CaCl_2$	3.11 ± 0.05	0.043 ± 0.001	72.8 ± 1.1
1.0 M NaCl / 40 mM $SrCl_2$	3.12 ± 0.02	0.041 ± 0.010	76.7 ± 2.0

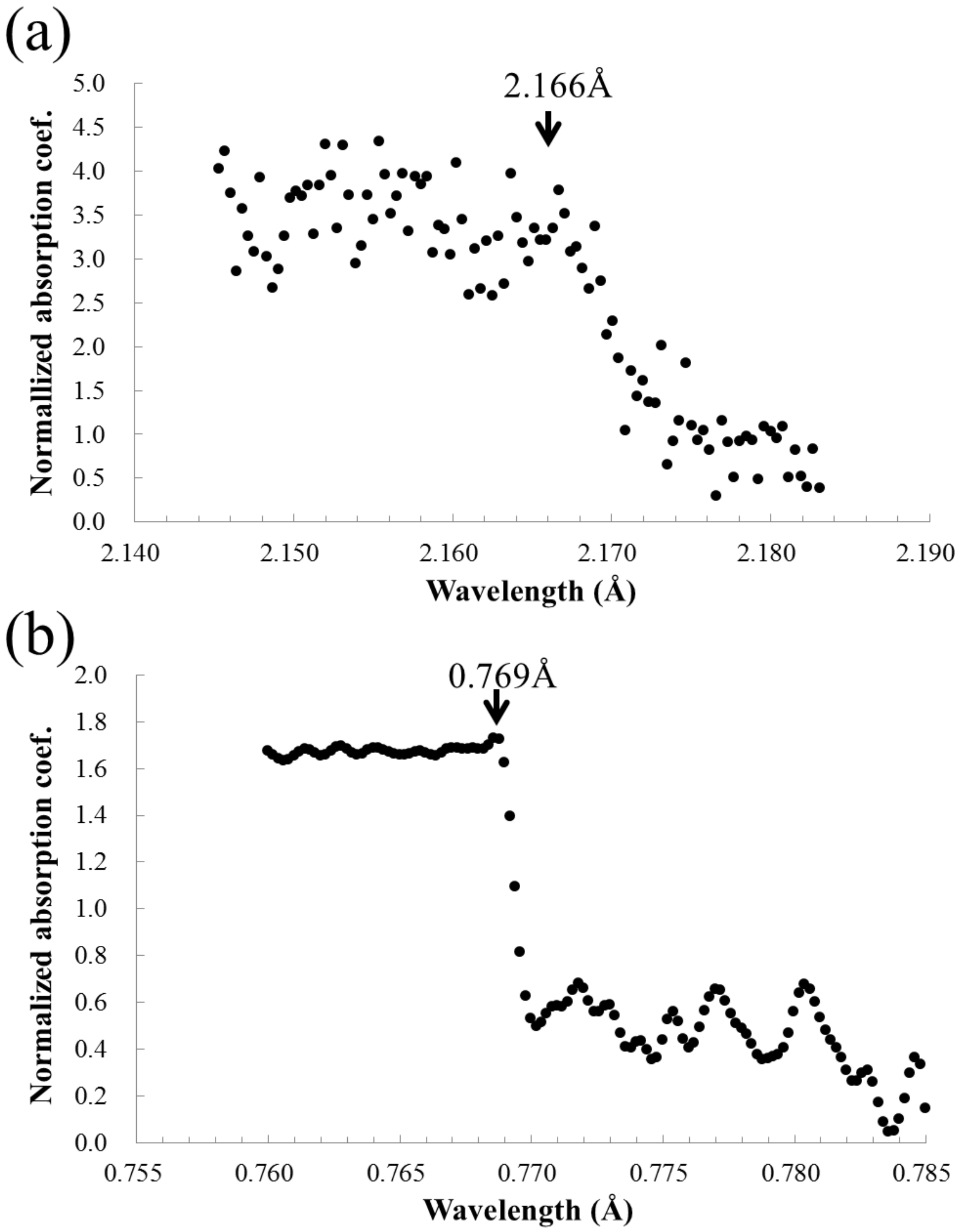

Figure S1 Examples of the X-ray absorption spectra of NQ-HaBLA crystals soaked in solutions containing (a) 100 mM Cs^{+} (condition-1A) and (b) 200 mM Sr^{2+} (condition-2A). These spectra (a) and (b) were obtained using BL7 at SAGA-LS and NW12A at PF, respectively. Arrows indicate the wavelength used for the X-ray anomalous diffraction data collection.
